# Correlations among satisfaction parameters after orthodontic treatment

**DOI:** 10.1590/2177-6709.29.5.e2424180.oar

**Published:** 2024-10-04

**Authors:** Eftychia LAMPRAKI, Fanouria PAPAIOANNOU, Ioulia-Maria MYLONOPOULOU, Nikolaos PANDIS, Iosif SIFAKAKIS

**Affiliations:** 1 National and Kapodistrian University of Athens, School of Dentistry (Athens, Greece).; 2 National and Kapodistrian University of Athens, School of Dentistry, Department of Orthodontics (Athens, Greece).; 3 University of Bern, School of Dentistry, Department of Orthodontics and Dentofacial Orthopedics (Bern, Switzerland).

**Keywords:** Orthodontic treatment, Patient satisfaction, Life satisfaction, Treatment outcome, Tratamento ortodôntico, Satisfação do paciente, Satisfação com a vida, Resultado do tratamento

## Abstract

**Objective:**

This study assessed patient’s satisfaction after orthodontic treatment in the postgraduate orthodontic clinic of the Dental School at the University of Athens (Athens, Greece), and investigated possible correlations between satisfaction after orthodontic treatment and life satisfaction, alongside the influence of age, gender, severity of initial orthodontic malocclusion and duration of retention period.

**Material and Methods:**

Patients aged 12 years and above, who had recently completed comprehensive orthodontic treatment, participated completing two questionnaires: a 37-item questionnaire, validated for assessing orthodontic treatment satisfaction; and the 5-item Satisfaction with Life Scale, a valid and reliable measure of life satisfaction. Scores from each questionnaire were summed for each patient. Demographic details, Index of Orthodontic Treatment Need (IOTN) and the duration of the retention period up to questionnaire completion were collected. Multiple regression analysis assessed the relationship between the questionnaires and the evaluated variables.

**Results:**

A total of 150 patients answered the questionnaires, being 82 women (55%) and 68 men (45%). The mean age of patients was 18.87 ± 5.97 years (range: 12-47). Most patients expressed satisfaction with treatment outcomes (grades 5 and 6). Satisfaction with orthodontic treatment showed a significant correlation with life satisfaction (*p*=0.002), but not with gender, age, dental/esthetic components of IOTN, or the duration of the retention period.

**Conclusion:**

Satisfaction after orthodontic treatment exhibited a significant correlation with life satisfaction, but it was not affected by gender, age, dental/esthetic components of IOTN, or the duration of the retention period.

## RESUMO

**Objetivo:**

Este estudo avaliou a satisfação dos pacientes após o tratamento ortodôntico, realizado na clínica de pós-graduação em Ortodontia da Faculdade de Odontologia da Universidade de Atenas (Atenas, Grécia), e investigou possíveis correlações entre a satisfação após o tratamento ortodôntico e a satisfação com a vida, juntamente com a influência da idade, sexo, gravidade da má oclusão inicial e duração da fase de contenção.

**Material e Métodos:**

Pacientes com 12 anos de idade ou mais, que haviam concluído recentemente tratamento ortodôntico, participaram preenchendo dois questionários: um questionário de 37 itens, validado para avaliar a satisfação com o tratamento ortodôntico; e a Escala de Satisfação com a Vida, de 5 itens, uma medida válida e confiável da satisfação com a vida. Os escores de cada questionário foram somados para cada paciente. Também foram coletados dados demográficos, Índice de Necessidade de Tratamento Ortodôntico (IOTN) e duração da fase de contenção até o preenchimento do questionário. Uma análise de regressão múltipla avaliou a relação entre os questionários e as variáveis estudadas.

**Resultados:**

No total, 150 pacientes preencheram os questionários, sendo 82 mulheres (55%) e 68 homens (45%). A média de idade dos pacientes foi de 18,87 ± 5,97 anos (variação: 12-47). A maioria dos pacientes expressou satisfação com os resultados do tratamento (escores 5 e 6). A satisfação com o tratamento ortodôntico apresentou correlação significativa com a satisfação com a vida (*p*=0,002), mas não com sexo, idade, componentes dentários/estéticos do IOTN ou duração da fase de contenção.

**Conclusão:**

A satisfação após o tratamento ortodôntico exibiu uma correlação significativa com a satisfação com a vida, mas não foi afetada pelo sexo, idade, componentes dentários/estéticos do IOTN ou pela duração da fase de contenção.

## INTRODUCTION

Dentofacial anomalies usually coexist with pronounced functional impediments, including masticatory, swallowing, and respiratory disturbances, as well as temporomandibular joint dysfunctions. Despite the prevailing functional issues, patients typically pursue therapeutic interventions primarily to enhance dentofacial aesthetics.^1^ In recent years, a significant increase in patients seeking orthodontic treatment has been observed. This is probably linked to the ever-increasing interest in their appearance and socioeconomic development.^2^ Several studies have focused on the motivations and expectations of patients seeking orthodontic treatment,^3^ ^,^ ^4^ but very few have investigated patient satisfaction and its affecting factors after completing orthodontic treatment.^2^ ^,^ ^5^ ^,^ ^6^ The success of orthodontic treatment is linked to the subjective improvement of facial and dental aesthetics; however, there are several objective criteria, such as: improvement in occlusion, maintenance of normal temporomandibular system function, dental and periodontal health, and long-term stability of the treatment outcome.^7^ Recognizing the benefits of treatment outcomes is crucial not only for specialized clinicians, but also for individuals undergoing orthodontic treatment.

Post-treatment satisfaction strongly hinges on aesthetic outcomes, which are subjectively perceived by patients themselves as offering psychological and social benefits such as changes in patients’ self-confidence.^8^ ^,^ ^9^ Subjective satisfaction level after patients’ orthodontic treatment can be regarded as an important parameter for measuring the overall outcome and significance of orthodontic treatment.^8^ ^,^ ^10^ ^,^ ^11^ Patient satisfaction is influenced by several factors, such as doctor-patient relationships,^2^ ^,^ ^8^ ^,^ ^12^ personality traits and age.^13^ ^-^ ^15^ Gender-based differences in post-treatment satisfaction are another subject of interest. While some studies suggested no significant differences,^5^ ^,^ ^9^ ^,^ ^16^ ^,^ ^17^ others proposed potentially lower satisfaction levels among females.^3^ ^,^ ^7^ The type of appliance used for retention influences patient satisfaction,^18^ as well as the dental office environment. A clean and well-structured dental office can result in a feeling of relaxation and comfort, contributing to patient satisfaction.^17^ On the other hand, dissatisfaction is associated with elevated pain levels and discomfort, difficulties in eating, gingivitis, and appliance breakage, leading the results of orthodontic treatment to be classified as unpleasant.^4^ ^,^ ^9^ ^,^ ^19^

A crucial objective of orthodontic treatment is to assist patients in achieving a great level of satisfaction with both their dentition and dental appearance.^13^ Thus, the aim of this study was to assess patient satisfaction after orthodontic treatment and patient satisfaction with life, in the postgraduate orthodontic clinic of the Dental School at the University of Athens (Athens, Greece). Secondary outcomes included the influence of age, sex, severity of initial malocclusion, and duration of the retention period on patient satisfaction.

## MATERIAL AND METHODS

The present study was a descriptive survey on patient satisfaction after orthodontic treatment, using two questionnaires. The protocol of the study was approved (136401, 15/12/2022) by the Ethics Committee of the School of Dentistry, National and Kapodistrian University of Athens (NKUA, Greece). The study was conducted on patients who had completed orthodontic treatment during the previous year and were treated by residents at the Department of Orthodontics, University of Athens, in which diverse urban and rural populations are treated, being selected to provide a varied ‘real-world’ sample. The following eligibility criteria were applied: patients aged over 12 years, who had completed their orthodontic treatment with fixed appliances on both arches during the previous year, and whose initial and final diagnostic data (medical and dental history, casts, photographs, radiographs) were of good quality. Patients with craniofacial deformities, syndromes, clefts, marked facial asymmetries, and marked functional deviation or protrusion were excluded.

**Figure 1:**
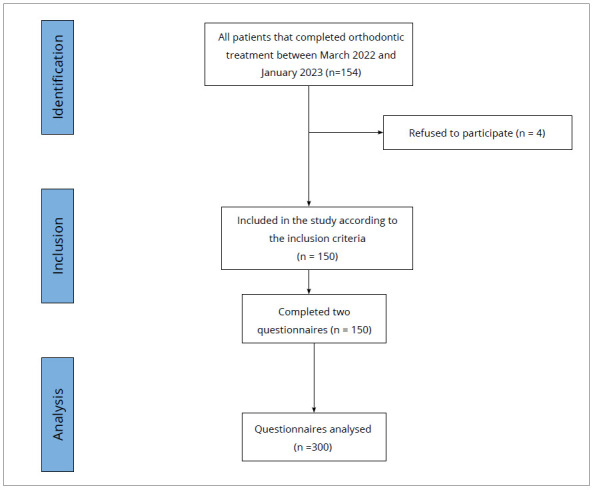
STROBE flow diagram.

The duration of the study was nine months, and all patients who completed their orthodontic treatment with fixed appliances during the previous year in the Department of Orthodontics, Dental School of Athens/NKUA, and met the inclusion criteria were recruited. The questionnaires were completed at their first retention check-up after appliance removal, at the end of the appointment, in a quiet room with adequate lighting and under the discretionary supervision of the investigator. Four patients refused to participate in the survey for personal reasons (Fig. 1). Informed consent was obtained from parents/legal guardians, and written consent was obtained from underage patients.

The questionnaires were administered as follows:

1) The Satisfaction with Life Scale (SWLS), which consists of five statements and is suitable for various age groups and applications^20^ (Q1, Supplementary data). Responses were marked on a scale from 1 (strongly disagree) to 7 (strongly agree), with score 4 being a neutral point. The SWLS has a possible range of scores of 5-35, with a score of 20 representing a neutral point on the scale. Scores between 5 and 9 indicate that the respondent is extremely dissatisfied with life, whereas scores between 31 and 35 indicate that the respondent is extremely satisfied. The coefficient alpha for the scale ranged from 0.79 to 0.89, indicating that the scale has high internal consistency. The scale was also found to have good test-retest correlations (0.84, 0.80 over a one-month interval).^21^
2) The satisfaction after orthodontic treatment questionnaire consisted of 37 questions^6^ (Q2, Supplementary data). Responses were marked on a scale of 1 (strongly disagree) to 6 (strongly agree), with no neutral points. An additional comment box was included at the end of the questionnaire for patient feedback and comments regarding the orthodontic treatment performed. The questionnaire has already been checked for internal consistency. The overall scale is best interpreted as a single dimension measuring ‘Patient Satisfaction’.^6^

The questionnaires were inspected for missing data, immediately after completion, by the main investigator.

Demographic data (age, sex) were collected for each patient, as well as the initial malocclusion, based on the dental and aesthetic components of IOTN (Index of Orthodontic Treatment Need), and the duration of the retention period. Twenty patients were randomly selected to complete the two questionnaires again after two weeks, to assess test-retest reliability - blank questionnaires and envelopes/stamps were given to each patient in this group, with a request for the patients to complete and return them after two weeks.

Every year, an estimated 200 patients finish their orthodontic treatment in the postgraduate clinic of the Department of Orthodontics, University of Athens, NKUA. According to recent research,^22^ if orthodontic appliances were removed in 200 patients in the last year, the required sample size should be 132 (*p*variance 50%, 95% confidence interval, 5% margin of error). A larger initial sample size was chosen to avoid selection bias and to allow statistical control for confounding variables.

### STATISTICAL ANALYSIS

The frequencies of the responses per question were calculated, and a sum score per patient across all questions for each questionnaire was obtained. Univariable nonparametric bootstrap (500 repetitions) regression models were fit to examine the effect of satisfaction with life, age, duration of the retention period, sex, and the esthetic and dental components of the IOTN. All significant variables from the univariable analyses and sex as an *a priori* confounder were entered into the final model. In addition, descriptive statistics were calculated after grouping the 37 questions into four domains pertaining to satisfaction with one’s appearance (Q1, Q3-7, Q22) and treatment area/staff (Q20, Q23-37), as well as the impact on psychology (Q8-15) and oral functions (Q2, Q16-19, Q21). All analyses were run using Stata 18 (Stata Corp, TX, USA). Cohen’s Kappa coefficient was used to evaluate test-retest reliability and was calculated for all questions individually.

## RESULTS

One hundred and fifty (n=150) patients answered the questionnaires, being 82 (55%) women and 68 (45%) men. The mean age of the patients was 18.87 years (SD: 5.97, min.-max.: 12-47 years). The mean treatment duration was 29.4 months (SD: 7.3). Cohen’s Kappa coefficient was used to evaluate test-retest reliability and was calculated for all questions individually. For Q1, the values ranged from 0.75 to 1.00. The lowest kappa (0.75) was observed for question #3 (*“I am satisfied with my life“*). For Q2, the values ranged from 0.45 to 1.00. The lowest Kappa (0.45) was observed for question #36 (*“The orthodontist treated me with respect”*).

**Figure 2:**
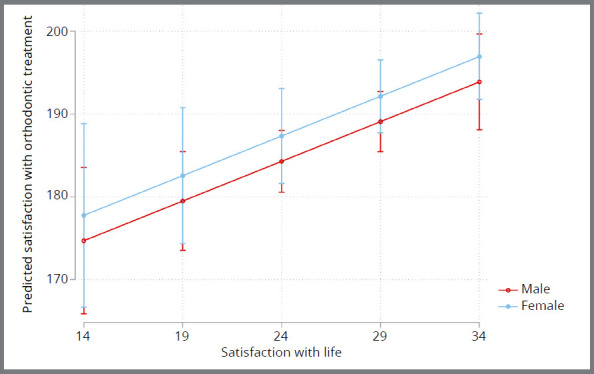
Adjusted predictions of satisfaction with orthodontic treatment as a function of satisfaction with life across genders.

The results of the regression models are presented in Table 1. Most patients were satisfied with the treatment. According to the univariable models, satisfaction with orthodontic treatment was significantly associated with life satisfaction (*p*=0.002), but not with age, sex, IOTN component or duration of the retention period. In the final model, only satisfaction with life remained significant. For every unit increase in satisfaction with life, an increase in satisfaction with orthodontic treatment of 0.96 units was observed (coef: 0.96, 95% CI: 0.33-1.59, *p*=0.003) after adjusting for sex. To make this more meaningful for every 10-unit increase in satisfaction with life, an increase of 9.6 units in satisfaction with orthodontic treatment is estimated (Fig. 2, Tables 1 and 2).

**Table 1:** Regression model results.

Variable	Coef. (95% conf. Interval)		p-value	Coef. (95% conf. Interval)	p-value
Satisfaction with life	Per unit	1.01 (0.37, 1.64)	0.002	0.96 (0.33, 1.59)	0.003
Age	Per unit	0.26 (-0.21, 0.72)	0.28		
Duration of retention period	Per unit	0.23 (-1.17, 1.73)	0.77		
IOTN Dental component	Per unit	0.95 (-2.46, 4.37)	0.59		
IOTN Esthetic component	Per unit	0.79 (-1.18, 2.76)	0.43		
Gender	Male	Reference			0.32
	Female	4.3 (-1.51, 10.15)	0.15	3.01 (-2.99, 9.12)	

**Table 2:** Median and interquartile range for Q2 subgroups.

Variable		n	p50	IQR
Q1, Q3-7, Q22	Satisfaction from appearance	150	36	2
Q20, Q23-37	Satisfaction from staff and treatment area	150	88	6
Q8-15	Psychological impact	150	38	10
Q2, Q16-19, Q21	Oral functions	150	32	7

Almost 19% of the patients gave the highest rating (strongly agree, rating #7) in the Q1 questionnaire (satisfaction with life), while 38% gave the second highest rating (agree, rating #6) (Suppl. Table 1). Regarding the first subgrouping in Q2 (satisfaction from appearance), 61% of the patients gave the highest rating (rating #6), and 17% gave the second highest rating (rating #5). Regarding the second subgrouping (satisfaction from treatment area/staff), 69% and 17% of the patients answered with a #6 or #5 rating, respectively. Regarding the third subgrouping (impact on psychology), the corresponding percentages were 37% and 24%, and the corresponding percentages for the fourth subgrouping (oral functions) were 49% and 27% (Suppl. Tables 2-5). Two patients added individual comments at the end of the questionnaire. According to their statements, they were satisfied overall with the orthodontic treatment received; however, waiting time and infrastructure should be improved.

## DISCUSSION

The present study aimed to assess satisfaction with orthodontic outcomes and with life within the first year after orthodontic treatment in a Greek population aged 12 years or older, who completed orthodontic treatment in a postgraduate clinic. Satisfaction after orthodontic treatment exhibited a significant correlation with life satisfaction. The questionnaire used to assess patient satisfaction after treatment was checked for validity, readability, and repeatability. This questionnaire was validated to assess orthodontic treatment satisfaction in a population aged 12-15 years after the completion of fixed orthodontic treatment. The readability level was deemed suitable for patients aged 15 years or older.^6^ A recent systematic review assessing the quality of patient satisfaction questionnaires relating to oral healthcare revealed methodological deficiencies, such as lack of consideration for populations other than adults, lack of internal consistency reporting of subscales within the questionnaire, and limited assessment of the stability of satisfaction scores over time.^23^ In the present study, these common methodological deficiencies were addressed: the population comprised both adults and adolescents, no subscales were used, and retesting was used for part of the sample (two weeks later), which may reflect a time of adjustment in their perception of the outcome of treatment.

According to the responses obtained, patients expressed a high level of satisfaction with the orthodontic treatment process and its outcomes. This noteworthy finding aligns with similar conclusions drawn in recent studies indicating that patients were satisfied to the extent of recommending the treatment to their friends.^4^ ^,^ ^5^ The present survey also evaluated the orthodontist-patient relationship throughout the treatment, revealing its significance in influencing patient satisfaction. Clear and regular explanations emerged as crucial elements that not only satisfied patients, but also contributed to an improved understanding of the treatment progress. Patients conveyed complete satisfaction with the orthodontists and the clinic staff and treatment area, emphasizing professionalism, a friendly environment, and confidence in the clinician’s competence; these results are consistent with the literature.^12^ ^,^ ^24^ Consequently, clinicians and departments must uphold elevated standards of patient satisfaction, to promote positive experiences. The questions to which patients responded less enthusiastically were those relating school performance and career to their orthodontic treatment. This highlights an area where further attention and communication may be beneficial to address potential concerns and enhance the overall patient experience. Overall, the waiting time in the present study was not an important factor for patient satisfaction. In contrast, a recent survey concluded that waiting time was not negligible for satisfaction with treatment.^17^

The analysis of the demographic data revealed that satisfaction with orthodontic treatment was not significantly correlated with variables such as age, IOTN score/malocclusion severity, or duration of the retention period. No discernible differences were noted between genders in terms of satisfaction with the treatment outcome. This result diverges from previous investigations,^3^ ^,^ ^7^ ^,^ ^25^ which consistently indicated higher satisfaction levels among male patients; but is similar to others^5^ ^,^ ^9^ ^,^ ^16^ ^,^ ^17^ that did not establish a significant correlation between gender and satisfaction with orthodontic outcomes. Although the present study did not identify significant age-related differences, existing research has reported a decline in satisfaction with dentofacial appearance with advancing age. Specifically, older patients expressed greater dissatisfaction with their dentofacial appearance and exhibited greater expectations for improvements in self-image and appearance than did their younger counterparts.^1^ ^,^ ^26^ This difference could stem from contemporary societal shifts and the evolving perspectives of adolescents, which are influenced significantly by social media. Both adolescents and adults are currently increasingly conscious of their appearance in this context.

In the present study, we sought to infuse innovation by exploring the connection between satisfaction after orthodontic treatment and overall life satisfaction. The SWLS, which has been used to quantify life satisfaction, has demonstrated its efficacy by not only correlating with measures of mental health, but also serving as a predictor of future behaviors on psychometry.^20^ ^,^ ^21^ The present results revealed a significant correlation between general life satisfaction and satisfaction after orthodontic treatment. This study may help orthodontists become aware of the potential implications of mental well-being for patient satisfaction. When dealing with patients who are dissatisfied with their lives or who have mental problems, there are several key aspects to which they should be aware: effective communication in a nonjudgmental manner, being sensitive to the patient’s emotional state, and actively listening to their concerns can help establish trust, establishing a network of referral sources and maintaining open communication with psychologists or counselors, for comprehensive patient care, ensuring that patient information is handled with privacy and in compliance with relevant healthcare regulations, and being aware of potential triggers and stress points during treatment. By incorporating these considerations into their practice, orthodontists can contribute to the well-being of their patients and provide a more supportive and understanding environment for those facing mental health challenges.

While the present survey presents promising data, several limitations should be acknowledged to provide a comprehensive interpretation of the findings. First, it is important to note that the results are based on subjects receiving orthodontic treatment at a university clinic, and as such, generalizing these findings to all orthodontic patients should be done with caution. Another notable limitation is the timeframe within which the questionnaire was administered - specifically, within the first year post-treatment. Typically, patients express greater enthusiasm about treatment outcomes immediately or shortly after completion.^6^ ^,^ ^9^ ^,^ ^25^ However, over an extended period, potential relapse may impact satisfaction levels. Future research addressing this limitation by extending the post-treatment assessment period could provide a more detailed understanding of patient satisfaction dynamics. Furthermore, the present study faced challenges in achieving a balanced representation of men and women in the sample, so future research could benefit from addressing this gender imbalance to enhance the robustness and generalizability of the findings. In addition, while other studies have shown variations in patients’ self-perception of dentofacial appearance and satisfaction based on ethnic backgrounds,^25^ ^,^ ^27^ this factor was not included in the present analysis. Race and ethnicity can be important variables in healthcare research, as they may influence health outcomes, access to healthcare, and responses to treatments. In future research, it could be beneficial to collect and analyze data related to race and ethnicity, with careful consideration of ethical and cultural sensitivity. Nevertheless, the association demonstrated in the present study may not be causal, since a weakness of cross-sectional studies is the inability to establish a causal relationship. Moreover, it was not possible to include an untreated control group, since this department does not have a wait list.

Notably, previous studies have highlighted disparities in the perception of occlusal problems between patients and orthodontists.^1^ ^,^ ^28^ ^,^ ^29^ Acknowledging and incorporating the patient’s perspective is vital for building a strong treatment relationship. Orthodontists should strive to gain a comprehensive understanding of how patients perceive their occlusal issues before initiating treatment.

## CONCLUSION

In conclusion, the patients expressed a high level of satisfaction with the orthodontic treatment process and its outcomes. A significant correlation was established between general life satisfaction and satisfaction after orthodontic treatment. Age, sex, initial malocclusion severity, and duration of the retention period were not significantly correlated with satisfaction after orthodontic treatment. This study also reinforces the importance of effective communication and positive orthodontist-patient relationships.

## Supplementary data (Q1): Satisfaction With Life Scale (SWLS), questionnaire to measure patients’ satisfaction with life.

[Fig f1a]

## Supplementary data (Q2): Questionnaire to measure patients’ satisfaction after orthodontic treatment.

[Fig f2a]

## Supplementary Table 1: Descriptive statistics for Q1 scale (satisfaction with life).

[Table t1a]

**Table t1a:**

Q1, satisfaction from life scale								
variable	values							Total
	1	2	3	4	5	6	7	
p1	0	1	4	21	24	77	23	150
	0.00	0.67	2.67	14.00	16.00	51.33	15.33	100.00
p2	0	4	1	22	28	61	34	150
	0.00	2.67	0.67	14.67	18.67	40.67	22.67	100.00
p3	0	3	2	8	23	70	44	150
	0.00	2.00	1.33	5.33	15.33	46.67	29.33	100.00
p4	0	10	7	32	30	46	25	150
	0.00	6.67	4.67	21.33	20.00	30.67	16.67	100.00
p5	11	17	10	38	23	35	16	150
	7.33	11.33	6.67	25.33	15.33	23.33	10.67	100.00
Total	11	35	24	121	128	289	142	750
	1.47	4.67	3.20	16.13	17.07	38.53	18.93	100.00

## Supplementary Table 2: Descriptive statistics for Q2 (satisfaction after orthodontic treatment) were calculated after grouping the 37 questions in four domains pertaining to satisfaction from appearance.

[Table t2a]

**Table t2a:**

Q2, first subgroup: satisfaction from appearance							
Question	Values						Total
	1	2	3	4	5	6	
q1	0	1	0	4	12	133	150
	0.00	0.67	0.00	2.67	8.00	88.67	100.00
q3	1	2	4	20	49	74	150
	0.67	1.33	2.67	13.33	32.67	49.33	100.00
q4	77	51	17	5	0	0	150
	51.33	34.00	11.33	3.33	0.00	0.00	100.00
q5	1	4	6	12	52	75	150
	0.67	2.67	4.00	8.00	34.67	50.00	100.00
q6	1	2	0	2	12	133	150
	0.67	1.33	0.00	1.33	8.00	88.67	100.00
q7	1	0	1	7	36	105	150
	0.67	0.00	0.67	4.67	24.00	70.00	100.00
q22	0	0	0	9	22	119	150
	0.00	0.00	0.00	6.00	14.67	79.33	100.00
Total	81	60	28	59	183	639	1,050
	7.71	5.71	2.67	5.62	17.43	60.86	100.00

## Supplementary Table 3: Descriptive statistics for Q2 (satisfaction after orthodontic treatment) were calculated after grouping the 37 questions in four domains pertaining to satisfaction from staff and treatment area.

[Table t3a]

**Table t3a:**

Q2, second subgroup: satisfaction from staff and treatment area							
Question	values						Total
	1	2	3	4	5	6	
q8	2	1	1	7	40	99	150
	1.33	0.67	0.67	4.67	26.67	66.00	100.00
q9	2	1	3	21	47	76	150
	1.33	0.67	2.00	14.00	31.33	50.67	100.00
q10	1	4	3	24	47	71	150
	0.67	2.67	2.00	16.00	31.33	47.33	100.00
q11	9	17	23	43	34	24	150
	6.00	11.33	15.33	28.67	22.67	16.00	100.00
q12	30	30	26	32	18	14	150
	20.00	20.00	17.33	21.33	12.00	9.33	100.00
q13	16	14	23	31	34	32	150
	10.67	9.33	15.33	20.67	22.67	21.33	100.00
q14	3	12	20	35	37	43	150
	2.00	8.00	13.33	23.33	24.67	28.67	100.00
q15	5	3	5	22	31	84	150
	3.33	2.00	3.33	14.67	20.67	56.00	100.00
Total	68	82	104	215	288	443	1,200
	5.67	6.83	8.67	17.92	24.00	36.92	100.00

## Supplementary Table 4: Descriptive statistics for Q2 (satisfaction after orthodontic treatment) were calculated after grouping the 37 questions in four domains pertaining to psychological impact.

[Table t4a]

**Table t4a:**

Q2, third subgroup: impact on psychology							
Question	Values						Total
	1	2	3	4	5	6	
q2	1	2	3	27	42	75	150
	0.67	1.33	2.00	18.00	28.00	50.00	100.00
q16	3	5	10	30	39	63	150
	2.00	3.33	6.67	20.00	26.00	42.00	100.00
q17	5	3	9	22	45	66	150
	3.33	2.00	6.00	14.67	30.00	44.00	100.00
q18	3	5	10	30	39	63	150
	2.00	3.33	6.67	20.00	26.00	42.00	100.00
q19	0	2	4	13	28	103	150
	0.00	1.33	2.67	8.67	18.67	68.67	100.00
q21	2	3	3	16	51	75	150
	1.33	2.00	2.00	10.67	34.00	50.00	100.00
Total	14	20	39	138	244	445	900
	1.56	2.22	4.33	15.33	27.11	49.44	100.00

## Supplementary Table 5: Descriptive statistics for Q2 (satisfaction after orthodontic treatment) were calculated after grouping the 37 questions in four domains pertaining to oral function.

[Table t5a]

**Table t5a:**

Q2, fourth subgroup: oral functions							
Question	Values						Total
	1	2	3	4	5	6	
q20	73	45	22	5	5	0	150
	48.67	30.00	14.67	3.33	3.33	0.00	100.00
q23	0	0	5	13	38	94	150
	0.00	0.00	3.33	8.67	25.33	62.67	100.00
q24	0	0	3	8	25	114	150
	0.00	0.00	2.00	5.33	16.67	76.00	100.00
q25	4	6	16	31	41	52	150
	2.67	4.00	10.67	20.67	27.33	34.67	100.00
q26	1	2	8	25	49	65	150
	0.67	1.33	5.33	16.67	32.67	43.33	100.00
q27	0	0	0	3	17	130	150
	0.00	0.00	0.00	2.00	11.33	86.67	100.00
q28	0	0	2	8	24	116	150
	0.00	0.00	1.33	5.33	16.00	77.33	100.00
q29	0	0	3	9	58	80	150
	0.00	0.00	2.00	6.00	38.67	53.33	100.00
q30	0	0	1	5	13	131	150
	0.00	0.00	0.67	3.33	8.67	87.33	100.00
q31	0	2	2	13	36	97	150
	0.00	1.33	1.33	8.67	24.00	64.67	100.00
q32	0	1	0	2	16	131	150
	0.00	0.67	0.00	1.33	10.67	87.33	100.00
q33	0	0	1	3	28	118	150
	0.00	0.00	0.67	2.00	18.67	78.67	100.00
q34	0	1	0	7	28	114	150
	0.00	0.67	0.00	4.67	18.67	76.00	100.00
q35	0	0	0	2	11	137	150
	0.00	0.00	0.00	1.33	7.33	91.33	100.00
q36	0	1	0	1	8	140	150
	0.00	0.67	0.00	0.67	5.33	93.33	100.00
q37	0	1	0	2	16	131	150
	0.00	0.67	0.00	1.33	10.67	87.33	100.00
Total	78	59	63	137	413	1,650	2,400
	3.25	2.46	2.62	5.71	17.21	68.75	100.00
